# Elevated maternal lipids in early pregnancy are not associated with risk of intrapartum caesarean in overweight and obese nulliparous women

**DOI:** 10.1186/1471-2393-13-143

**Published:** 2013-07-09

**Authors:** Elaine M Fyfe, Karen S Rivers, John MD Thompson, Kamala PL Thiyagarajan, Katie M Groom, Gustaaf A Dekker, Lesley ME McCowan

**Affiliations:** 1 Department of Obstetrics and Gynaecology, University of Auckland, Private Bag 92019, Auckland, New Zealand; 2 Women and Children’s Division, Lyell McEwin Hospital, University of Adelaide, Adelaide, Australia; 3 Department of Paediatrics, University of Auckland, Private Bag 92019, Auckland, New Zealand; 4 Department of Obstetrics and Gynaecology, Faculty of Medical and Health Science, University of Auckland, Private Bag 92019, Auckland, New Zealand

**Keywords:** Cholesterol, Antenatal, Delivery, Obesity, Labour

## Abstract

**Background:**

Maternal overweight and obesity are associated with slower labour progress and increased caesarean delivery for failure to progress. Obesity is also associated with hyperlipidaemia and cholesterol inhibits myometrial contractility in vitro. Our aim was, among overweight and obese nulliparous women, to investigate 1. the role of early pregnancy serum cholesterol and 2. clinical risk factors associated with first stage caesarean for failure to progress at term.

**Methods:**

Secondary data analysis from a prospective cohort of overweight/obese New Zealand and Australian nullipara recruited to the SCOPE study. Women who laboured at term and delivered vaginally (n=840) or required first stage caesarean for failure to progress (n=196) were included. Maternal characteristics and serum cholesterol at 14–16 weeks’ of gestation were compared according to delivery mode in univariable and multivariable analyses (adjusted for BMI, maternal age and height, obstetric care type, induction of labour and gestation at delivery ≥41 weeks).

**Results:**

Total cholesterol at 14–16 weeks was not higher among women requiring first stage caesarean for failure to progress compared to those with vaginal delivery (5.55 ± 0.92 versus 5.67 ± 0.85 mmol/L, *p=* 0.10 respectively). Antenatal risk factors for first stage caesarean for failure to progress in overweight and obese women were BMI (adjusted odds ratio [aOR (95% CI)] 1.15 (1.07-1.22) per 5 unit increase, maternal age 1.37 (1.17-1.61) per 5 year increase, height 1.09 (1.06-1.12) per 1cm reduction), induction of labour 1.94 (1.38-2.73) and prolonged pregnancy ≥41 weeks 1.64 (1.14-2.35).

**Conclusions:**

Elevated maternal cholesterol in early pregnancy is not a risk factor for first stage caesarean for failure to progress in overweight/obese women. Other clinically relevant risk factors identified are: increasing maternal BMI, increasing maternal age, induction of labour and prolonged pregnancy ≥41 weeks’ of gestation.

## Background

Caesarean delivery in overweight and obese women is associated with increased morbidity and mortality, along with increased utilisation of health care resources [1]. Increasing maternal body mass index (BMI) is associated with a dose dependent elevated risk of emergency caesarean section in labour, which is largely due to failure to progress [1-5]. We have previously demonstrated that this elevated risk is confined to the first stage of labour, and rates of caesarean in the second stage of labour do not differ according to BMI [4].

Factors that differentiate overweight and obese women who deliver vaginally from those who require intrapartum caesarean have not previously been reported. The ability to identify those overweight and obese women in early pregnancy who are at higher risk for caesarean in labour at term might assist with delivery planning and counselling.

The underlying reason for failure to progress is thought to be reduced uterine contractility [5]. Reduced contractility in term myometrial biopsies has been reported in specimens from overweight and obese women compared with those from women of normal weight [5]. In addition the rate of cervical dilation in labour is slower in overweight and obese women [6]. However, the mechanisms underlying the association between elevated BMI and caesarean section for failure to progress in overweight and obese women are not understood. The relative hyperlipidaemia of normal pregnancy is exaggerated in obese women [7] and it has been speculated that obesity related dyslipidaemia may contribute to altered myometrial cell signalling, dysfunctional labour and increased caesarean delivery for failure to progress [8].

Among overweight and obese women, the level of serum cholesterol may differentiate those who display impaired myometrial function and require caesarean section in labour from those who progress to a vaginal birth. This association has not previously been investigated.

We aimed to investigate the relationship between maternal characteristics, including serum cholesterol, and the risk of caesarean for failure to progress in the first stage of labour among overweight and obese women. We hypothesised that overweight and obese nulliparous women at term who required a caesarean in the first stage of labour for failure to progress would have higher serum cholesterol levels at 14–16 weeks’ of gestation compared with those who had a vaginal delivery.

## Methods

**Figure 1:**
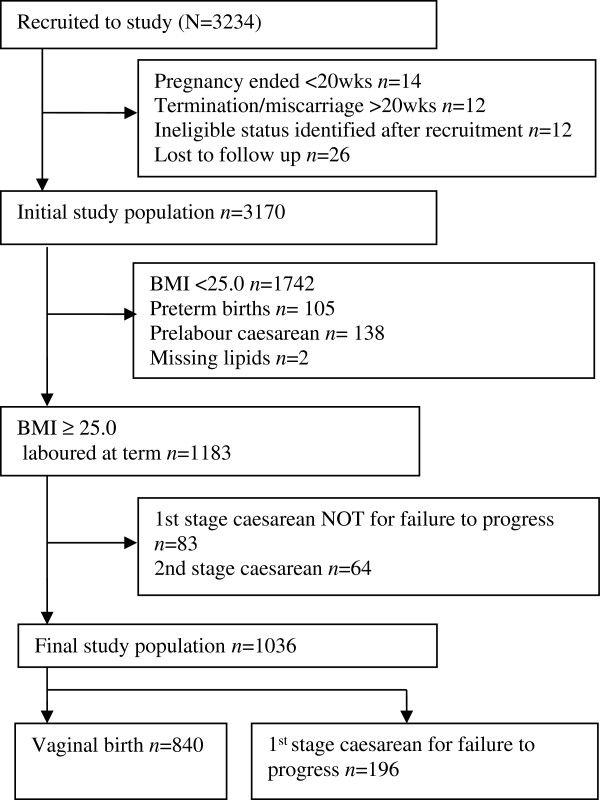
Recruitment flow chart.

Participants were healthy, nulliparous women recruited to the Screening for Pregnancy Endpoints Study (SCOPE) from Auckland, New Zealand and Adelaide, Australia [9]. This is a secondary analysis of data from the SCOPE study, a multicentre prospective cohort study with the primary aim of developing screening tests for prediction of preeclampsia, spontaneous preterm birth and small for gestational age babies. Ethical approval was obtained from local ethics committees (New Zealand AKX/02/00/364, Australia REC 1712/5/2008) and all women provided written informed consent. The population for the current study comprised overweight and obese women who laboured at term (Figure 1) and either delivered vaginally (spontaneous or operative) or had a caesarean in the first stage of labour for failure to progress. Women who had a caesarean in the first stage of labour for any reason other than failure to progress or had a caesarean in the second stage of labour were excluded.

Detailed data collected from all participants during interview with a research midwife, at 14–16 weeks gestation, have previously been described in detail [10] and included demographic information, smoking, family, medical and gynaecological history. Maternal measurements recorded at 14–16 weeks of gestation included blood pressure, height and weight. Maternal BMI was calculated at 14-16weeks’ of gestation using maternal weight divided by height squared (kg/m^2^) measured to the nearest kilogram and centimetre respectively. Overweight and obesity were defined according to conventional WHO criteria as BMI 25–29.99 and ≥ 30 kg/m^2^ respectively [11].

Non-fasting serum cholesterol was measured from bloods drawn at the time of interview at 14–16 weeks’ of gestation in the SCOPE study. Blood specimen collection and preparation of serum were performed using standard operating procedures, and samples were stored at −80°C within four hours of collection. Measurements were performed in two batches in 2008. Samples were measured using the cobas® Cholesterol CHOD-PAP kit (Roche/Hitachi). Co-efficient of variation was 0.8% and 1.7% for within and between-run respectively.

### Outcome measures and exposures

The primary outcome measure was mode of delivery classified as vaginal [spontaneous or operative (forceps or ventouse)] or caesarean in first stage of labour for failure to progress. The exposure was non-fasting serum cholesterol level measured at 14–16 weeks of gestation. Cholesterol measurements (mmol/L) included total cholesterol, high density lipoprotein (HDL)-cholesterol, low density lipoprotein (LDL)-cholesterol, and total cholesterol: HDL-cholesterol ratio.

### Definitions

Estimated date of delivery was calculated from a certain last menstrual period date and only adjusted if either an ultrasound scan at less than 16 weeks’ gestation found a difference of seven or more days between the scan gestation and that calculated by the last menstrual period, or at a 20 week scan a difference of 10 or more days was found between the scan gestation and that calculated from the last menstrual period. If the last menstrual period date was uncertain, scan dates were used to calculate the estimated date of delivery [9]. Socioeconomic index was a measure of socioeconomic status derived from maternal occupation [12]. Private obstetric care was that provided by a private obstetrician and the comparison group included care that was hospital based, or provided by an independent midwife or a general practitioner. Definitions for gestational hypertension and preeclampsia have previously been described in detail [10,13]. Term delivery was delivery at 37° or greater weeks of gestation. Active labour was defined as regular, painful uterine contractions with progressive cervical effacement and dilation and cervical dilatation ≥3 cms [14]. Emergency caesarean in labour was delivery required because of an emergency situation in active labour (e.g. failure to progress, fetal distress) when the caesarean was performed having not been previously considered necessary [15]. Indication for caesarean stated on the delivery summary by the attending doctor was used to determine whether caesarean in first stage was performed for failure to progress or another indication. Participating hospitals had standard protocols for management of dysfunctional labour in nulliparous women which included rupture of membranes and oxytocin augmentation which preceded diagnosis of failure to progress. If there were combined indications which included failure to progress, then the indication was recorded as failure to progress. Gestational Diabetes Mellitus was defined in accordance with The Australian Diabetes in Pregnancy Society [16]. Small for gestational age (SGA) and large for gestational age (LGA) were infants with birthweight <10^th^ customised centile and >90^th^ customised centile respectively, adjusted for infant sex, gestation at delivery and maternal characteristics: parity, ethnicity, height and booking weight [17].

### Statistical analysis

Data were entered into an internet accessed, auditable database *(Medscinet AB, Sweden).* Data analysis was performed using the statistical software package SAS version 9.2. Univariable analysis was performed to compare maternal characteristics and birth outcomes between women who had a vaginal delivery (spontaneous or operative) and women who had a first stage caesarean for failure to progress. Vaginal birth was the referent group. The chi square test was used for analysis of categorical variables, and a Student’s *t*-test was used to compare continuous variables. For continuous variables we investigated the shape of the relationship using Generalised Additive Models (GAMs) [18]. There were no missing data for any variables used in this analysis. Adjusted odds ratios were calculated using multivariable logistic regression, controlling for all variables significant at p<0.1 in univariable analyses, and all maternal cholesterol measures as a priori variables. With our sample size (N=1036, 840 vaginal and 196 caesarean births) we are able to detect a difference of 0.2 mmol/L in total cholesterol between the groups (alpha of 0.05 and power 80%). Therefore, we are able to detect any differences that would be considered clinically significant and which are greater than 0.2 mmols.

## Results

Between November 2004 and Oct 2008, 3234 women were recruited to the SCOPE study in Auckland and Adelaide and follow up was complete in 3195 (99%) participants (Figure 1). The initial study population (*n*=3170) included 1742 (55.0%) women with BMI<25 who were excluded from this study. Of the final eligible study population of 1036 nulliparous overweight and obese women who laboured at term, 840 (81.1%) had a vaginal delivery and 196 (18.9%) had a first stage caesarean for failure to progress. Of the women who had first stage caesarean for failure to progress, 53 (27%) also had fetal distress and therefore had a combined indication for caesarean delivery.

### Maternal characteristics and antenatal outcomes

Women who had a first stage caesarean for failure to progress were older, shorter, and had a higher BMI compared to those who delivered vaginally (Table 1). There were no differences in ethnicity, socioeconomic index, smoking, marital status or rates of gestational diabetes or hypertensive pregnancy disorders according to mode of delivery. Serum cholesterol levels at 14–16 weeks’ of gestation were not higher among overweight and obese women who had a first stage caesarean for failure to progress compared with overweight and obese women who delivered vaginally (Total cholesterol 5.67±0.85 v 5.55 ± 0.92, p=0.10) (Table 1). There were also no differences in other lipid parameters (Table 1).

**Table 1:** Maternal characteristics, pregnancy and neonatal outcomes for overweight and obese nulliparous women according to mode of delivery

	**Vaginal delivery**	**1**^**st **^**stage caesarean for failure to progress**	***P***
	**N = 840 (81%)**	**N = 196 (19%)**	
Maternal characteristic			
Body mass index (kg/m^2^) [median (IQR)]	28.0 (25.4-31.3)	29.7 (27.0-32.7)	<0.001
Age (years)	27.1 ± 5.6	28.9 ± 5.9	<0.001
Ethnicity			0.18
European	732 (87)	179 (91)	
Socioeconomic index	38 ± 16	39 ± 15	0.35
Smoking at 14–16 weeks	103 (12)	22 (11)	0.69
Unmarried	77 (9)	13 (7)	0.26
Maternal height (cm)	166 ± 6.4	163 ± 5.8	<0.001
Private obstetric care	83 (10)	28 (14)	0.07
Pregnancy complications			
Gestational diabetes*	26 (3)	12 (6)	0.11
Gestational hypertension	85 (10)	25 (13)	0.28
Preeclampsia	46 (5)	16 (8)	0.15
Serum lipids			
Total Cholesterol^†^	5.55 ± 0.92	5.67 ± 0.85	0.10
LDL^‡^- Cholesterol^†^	3.10 ± 0.82	3.18 ± 0.75	0.20
HDL^§^- Cholesterol^†^	1.75 ± 0.36	1.75 ± 0.34	0.87
Total Cholesterol: HDL ratio	3.29 ± 0.80	3.34 ± 0.76	0.42
Triglycerides	1.55 ± 0.63	1.62 ± 0.63	0.16
Labor and delivery			
Induction of labour	259 (31)	100 (51)	<0.001
Gestation at delivery (weeks)	39.6 ± 1.2	39.9 ± 1.2	0.002
Gestation at delivery ≥ 41 weeks	205 (24)	71 (36)	0.002
Neonatal outcomes			
Birthweight (g)	3500 ± 445	3734 ± 484	<0.001
Customised birthweight centile	44 ± 28	57 ± 29	<0.001
Small for gestational age^║^	91 (11)	12 (6)	0.05
Large for gestational age^║^	56 (7)	32 (16)	<0.001

Data are n (%) or mean ± standard deviation unless otherwise stated.

* Unknown n=40.

^†^ mmol/L.

^‡^ low density lipoprotein.

^§^ high density lipoprotein.

^║^By customised birthweight centiles.

### Labour and delivery outcomes

Of 1036 overweight and obese women at term, 677 (65%) laboured spontaneously, and 359 (35%) had labour induced. The rate of first stage caesarean for failure to progress following induction of labour was twice as high as that following spontaneous onset of labour (28% v 14%, *p*=<0. 001). Although women who had a first stage caesarean for failure to progress had an increased final gestation at delivery, the difference of only two days was not clinically significant (Table 1). Those with caesarean for failure to progress in the first stage of labour were more likely to have a prolonged pregnancy (≥ 41° weeks) compared to those who delivered vaginally. The mean customised birthweight centile and the rate of LGA infants was higher among women who had a first stage caesarean for failure to progress, and the rate of SGA infants was lower (Table 1).

Following adjustment for all variables in univariable analysis with *P* value <0.1, maternal total cholesterol, LDL, HDL and total LDL: HDL ratios were not independently associated with risk of first stage caesarean for failure to progress. The following variables were identified as independent antenatal risk factors for first stage caesarean for failure to progress: decreasing maternal height, increasing maternal age, increasing BMI, induction of labour and gestation at delivery greater than or equal to 41 weeks (Table 2). We investigated the interaction between BMI and maternal height in relation to caesarean delivery, and found that this relationship was not significant. As birthweight is not an antenatal risk factor, we did not include it as a variable in the final multivariable model. However, when a secondary analysis was undertaken that included birthweight, the risk for caesarean doubled for every 500 g increase in birthweight (aOR 2.19, 95% CI 1.78-2.69) and gestation at delivery ≥41 weeks was no longer significant (aOR 0.92, 95% CI 0.62-1.37). Hence, in our multivariable model of antenatal risk factors for caesarean, prolonged pregnancy (≥41 weeks) likely acts as a surrogate for increasing birthweight (Table 2).

**Table 2:** **Antenatal risk factors for 1**^**st **^**stage caesarean for failure to progress among overweight and obese nullipara**

**Antenatal factor**	**Adjusted odds ratio**
	**(95% confidence interval)**
BMI† (per 5 units ↑)	1.40 (1.18-1.66)
Maternal age (per 5 yr ↑)	1.37 (1.17-1.61)
Maternal height (per 1 cm ↓)	1.09 (1.06-1.12)
Induction of labour	1.94 (1.38-2.73)
Gestation at delivery ≥ 41 wks	1.64 (1.14-2.35)

† kg/ m^2^.

## Discussion and conclusions

In this cohort of overweight and obese nulliparous women who laboured at term we have demonstrated, contrary to our hypothesis, that those requiring first stage caesarean for failure to progress are not more likely to have higher serum cholesterol levels at 14–16 weeks’ of gestation compared with overweight and obese women who deliver vaginally. We have identified clinically relevant antenatal risk factors among overweight and obese women for first stage caesarean for failure to progress at term.

Our findings are novel as the relationship between maternal serum cholesterol and caesarean for failure to progress in overweight and obese women has not previously been described. We have previously demonstrated that overweight and obesity in nulliparous women confers an independent risk for caesarean only in the first stage and not in the second stage of labour [4]. In vitro studies using term myometrial biopsies from women undergoing intrapartum caesarean have demonstrated inhibited contractile amplitude following addition of cholesterol to the medium, leading to the postulation that higher serum cholesterol levels may contribute to sub-optimal myometrial contractility [19]. Oxytocin and oestrogen receptor function is modulated by the amount of cholesterol in the uterine myometrial plasma membranes and extraction of cholesterol from myometrial plasma membranes in vivo has been demonstrated to greatly enhance spontaneous contractions [20]. Although we did not find a difference in cholesterol levels in early pregnancy by mode of delivery, as cholesterol levels continue to increase with advancing gestation [21], it is possible that either late pregnancy cholesterol levels or the magnitude of increase might influence the risk of caesarean for failure to progress. It was not possible to explore this relationship in the current study as we did not collect late pregnancy samples in the SCOPE study where the focus was early pregnancy risk prediction. We used non fasting lipids for our study, however lipid profiles change minimally in response to food intake [22], and a very large recent study showed that fasting times showed little association with lipid subclass levels in a community-based population, suggesting that fasting for routine lipid levels is largely unnecessary [23]. Non fasting lipid profiles may be used as standard [24]. Our findings of several simple antenatal risk factors associated with caesarean for failure to progress specifically among overweight and obese nulliparous women are novel and clinically relevant. Although many studies have identified risk factors associated with caesarean for failure to progress in a general obstetric population of mixed BMI [25], and identified obesity as a risk factor, no studies have specifically investigated risk factors among overweight and obese women only. Two studies with populations of mixed BMI have performed subgroup analyses of obese women and reported a two to three fold increase in the rate of caesarean among obese women with short stature [26,27] but a third study reporting interventions during labour in relation to height in obese women did not support this association [28]. An association between induction of labour and caesarean delivery among nulliparous women of mixed BMI has previously been reported [29], particularly for labour arrest [30].

There are some limitations to our study. This study is a retrospective secondary analysis of prospectively collected data. We used a definition of regular, painful uterine contractions with progressive cervical effacement and dilation and cervical dilatation ≥3 cms for active labour and this was incorporated into our subsequent definition of failure to progress in active labour. There are several published definitions of active labour, however there is no consistently agreed consensus. Suggested criteria to diagnose active labour include cervical dilatation ranging from ≥2 cms [31] to ≥6 cms suggested in a recent publication [32]. However, this latter definition of ≥6 cms is applicable only to multiparous women, as in this study, the average labour curve for nulliparous women did not show a definitive inflection point at which labour progressed at an accelerated rate [32]. We selected our definition based both on the pre-defined definition in the SCOPE study protocol which was consistent with the definitions used in practice at the two participating centres. It is unlikely therefore we have introduced systematic bias. It is possible our finding of no effect of early pregnancy cholesterol on the risk of caesarean for failure to progress in the first stage may be related to our definition of active labour and subsequent diagnosis of failure to progress. Further research using an increased cervical dilatation for definition of active labour should be considered in future studies.

In summary, we have identified the following antenatal risk factors for first stage caesarean for failure to progress among overweight and obese nulliparous women at term: increasing maternal age, increasing BMI, reducing height, induction of labour and prolonged pregnancy. These factors can be incorporated into risk assessment when planning for delivery in overweight and obese women. Early pregnancy cholesterol measures are not useful as part of this risk assessment.

### Details of ethics approval

Ethical approval was obtained from local ethics committees (New Zealand AKX/02/00/364, Australia REC 1712/5/2008).

## Competing interests

The authors declare that they have no competing interests.

## Authors’ contributions

EF conceived of the study and participated in its design, performed the statistical analysis, interpreted the data and drafted and revised the manuscript. KR assisted in collecting the data and helped to draft and revise the manuscript. JT assisted with statistical analysis, interpreted the data, and helped to draft and revise the manuscript. KT assisted in collecting the data and helped to draft and revise the manuscript. KG helped to draft and revise the manuscript. GD helped to draft and revise the manuscript. LMcC conceived of the study and participated in its design, interpreted the data and helped to draft and revise the manuscript. All authors read and approved the final manuscript.

## Pre-publication history

The pre-publication history for this paper can be accessed here:

http://www.biomedcentral.com/1471-2393/13/143/prepub
